# Diagnostic and therapeutic strategies for vascular complications after renal transplantation: a single-center experience in 2,304 renal transplantations

**DOI:** 10.3389/frtra.2023.1150331

**Published:** 2023-05-11

**Authors:** Jiangwei Zhang, Wujun Xue, Puxun Tian, Jin Zheng, Chenguang Ding, Yang Li, Ying Wang, Xiaoming Ding

**Affiliations:** Department of Renal Transplantation, The First Affiliated Hospital of Xi‘an Jiaotong University, Xi'an, China

**Keywords:** renal transplantation, vascular complications, diagnostic and therapeutic strategies, percutaneous transluminal angioplasty, open surgery

## Abstract

Vascular complications after renal transplantation are one of the serious surgical complications, which can affect the transplantation outcome and even endanger life if not treated properly. We performed a retrospective analysis of the 2,304 renal transplantations procedures completed between the period of Jan., 2015 and Jan., 2022, which consisted of 1,658 male patients and 646 female patients. Among the above cases, there were 54 cases of vascular complications after renal transplantation, the incidence of vascular complications in our study was 2.34% (54/2,304), the most common vascular complication was transplanted renal artery stenosis (TRAS, *n* = 36), followed by external iliac artery dissection (*n* = 5), renal artery rupture (*n* = 4), renal vein thrombosis (*n* = 3), renal artery thrombosis (*n* = 2), renal artery dissection (*n* = 1), renal artery pseudoaneurysm (*n* = 1), and internal iliac artery pseudoaneurysm (*n* = 1), and renal artery kinking (*n* = 1). 40 patients were treated by percutaneous transluminal angioplasty (PTA), including 3 balloon catheter dilatation and 37 endovascular stentings, and 14 underwent open surgery. Eventually, 9 patients had graft nephrectomy, resulting in an overall treatment rate of 81.5%. Most vascular complications can be treated satisfactorily with PTA. However, the overall treatment of renal artery rupture, thrombosis, renal artery kinking, and other complications is poor, and the rate of transplanted renal loss is high.

## Introduction

Renal transplantation is currently the most effective approach for the treatment of end-stage renal disease, with a 1-year survival rate of the transplanted renal having increased to more than 95% after transplantation (1). The incidence of vascular complications after renal transplantation has decreased in recent years with the continuous technological advances in donor-recipient evaluation criteria, organ acquisition and preservation, renal transplantation surgery, and vascular anastomosis, with studies reporting an incidence of 1.35% (2). However, vascular complications after renal transplantation are still one of the serious surgical complications, especially some cases are characterized by insidious onset, rapid progression, and severe destruction, which not only result in direct functional impairment of the transplanted renal but also increases the risk of renal failure, and in severe cases even endangers life (3). A systematic retrospective analysis was conducted of the clinical data of 54 patients with vascular complications after renal transplantation and the efficacy of different treatment modalities applied to the patients admitted to the Department of Renal Transplantation, First Affiliated Hospital of Xi'an Jiaotong University from January, 2015 to January, 2022 was conducted to provide a referential basis for the clinical diagnosis and treatment of patients, so as to reduce the incidence of vascular complications, improve the transplantation outcome and survival rate.

## Materials and methods

A retrospective analysis was performed on 2,304 renal transplantations completed in the Department of Renal Transplantation of the First Affiliated Hospital of Xi'an Jiaotong University between January, 2015 and January, 2022, which consisted of 1,658 male patients and 646 female patients with an average age of 48.7 ± 15.6 years old (6–68 years old), 35 (1.5%) were children and the remaining 2,269 (98.5%) were adults. 54 cases of vascular complications after renal transplantation occurred during this period, including 48 cases of deceased donation (DD) renal transplantation and 6 cases of living renal transplantation. We used open surgery to remove the donor renal, with no damage to the renal arterial intima. All preoperative assessments and examinations were completed in accordance with the renal transplantation treatment protocol, and no significant contraindications to surgery were observed. The mean follow-up observation time for patients with vascular complications was 3.4 ± 2.65 years (10 months–7 years), and we defined early vascular complications after renal transplantation as the first month after transplantation and late complications as the first month to several years after transplantation. All patients underwent a classic open rectus abdominis posterolateral arcuate incision, a single donor renal artery (*n* = 43) anastomosed to the internal or external iliac artery, a double donor renal artery (*n* = 8) or multiple donor renal arteries (*n* = 3), which has an anastomosis with the internal iliac artery, external iliac artery or inferior abdominal wall artery, and all donor renal veins were anastomosed to the external iliac vein. The immunosuppressive protocol in this study were all formulated in accordance with the diagnostic and therapeutic specifications of the First Affiliated Hospital of Xi'an Jiaotong University. The perioperative induction regimens included the application of interleukin-2 receptor antagonist (IL-2Ra, basiliximab, *n* = 20) or anti-thymocyte globulin in layers according to the immune risk after renal transplantation. Namely, anti-human T lymphocyte rabbit immunoglobulin (ATG-F, *n* = 21) or rabbit anti-humanthymocyte immunoglobulin (rATG, *n* = 13) was used for immune induction. Maintenance oral immunosuppressive protocol: The basic immunosuppressive protocol initially used in renal transplantation recipients was the triple immunosuppressive protocol of calcineurin inhibitor (CNI)± mycophenolate mofetil (MMF)± prednisone (Pred). CNI included tacroclimus (Tac, *n* = 38) and cyclosporin A (CsA, *n* = 16). The patient data were obtained from the medical record, and all patients signed an information consent form, were informed about the study and agreed to have their clinical information used in the reported research. The study was reviewed and approved by the Ethics Committee of the First Affiliated Hospital of Xi'an Jiaotong University, no executed prisoners were used for any part of this study. Descriptive statistics are used for the percentage of discrete category.

## Results

A total of 54 patients in this study had vascular complications after renal transplantation (2.34%, 54/2,304), which is generally consistent with the literature. The most common vascular complication was transplanted renal artery stenosis (TRAS, *n* = 36), followed by external iliac artery dissection (*n* = 5), renal artery rupture (*n* = 4), renal vein thrombosis (*n* = 3), renal artery thrombosis (*n* = 2), renal artery dissection (*n* = 1), renal artery pseudoaneurysm (*n* = 1), internal iliac artery pseudoaneurysm (*n* = 1), and renal artery kinking (*n* = 1), Table 1. Among these 54 patients with vascular complications after renal transplantation, 40 patients were treated by percutaneous transluminal angioplasty (PTA), including 3 balloon catheter dilation and 37 endovascular stentings, and 14 underwent open surgical treatment, with 9 patients eventually had graft nephrectomy. Vascular complications occurred in 19 cases (35.2%) in the early postoperative period and in 35 cases (64.8%) in the late postoperative period. The main manifestations of early postoperative complications included acute deterioration of graft function, sudden decrease in urine output, hemorrhage, and graft loss, while late postoperative complications mostly showed mild symptoms and were not easily detected. 5 cases of renal artery stenosis occurred in the early postoperative period, 4 cases had stents placed, and one case had open surgery to remove the stenotic segment and reanastomose it. We found 31 cases of renal artery stenosis in the late postoperative period, including 28 cases with renal artery stents and balloon dilatation in 3 pediatric recipients, of which 25 cases presented with hypertension or graft renal insufficiency, and the remaining 3 cases had no obvious clinical manifestations and occurred only by routine postoperative follow-up ultrasound, and all cases were confirmed by selective angiography. 5 patients developed external iliac artery dissection, of which 4 were intra-operative external iliac artery dissection, including 3 treated with artificial vessel replacement, transplanted renal artery anastomosis with internal iliac artery after secondary perfusion of the transplanted renal, one treated with donor iliac artery bypass, and one occurred 3 months after surgery, and the covered stent was placed through interventional procedures, and 5 transplanted renal function eventually returned to normal. 4 patients were diagnosed with renal artery rupture, which occurred in the perioperative period, manifested as peri-transplant hematoma, abdominal pain, decreased blood pressure, etc. In 3 cases was diagnosed as a result of infectious arteritis, and the transplanted renal had to be removed due to irreparable infection, and in one patient was repaired due to loose sutures, and the function of the transplanted renal was restored to normal. Renal vein thrombosis (*n* = 3) and renal artery thrombosis (*n* = 2) both occurred in the perioperative period. 3 patients with renal vein thrombosis and one patient with renal artery thrombosis underwent surgical exploration, and all were unable to restore the function of the transplanted renal, so the transplanted renal was removed. One patient who developed renal artery thrombosis was treated with PTA, thrombolysis, and intraluminal stent placement, and postoperative delayed graft function (DGF) occurred, and renal function returned to normal after 2 weeks. One patient developed renal artery dissection 3 days after surgery, and the normal function of the transplanted renal was restored by interventional surgery to place a membrane-covered stent. One patient had a renal artery pseudoaneurysm 3 months after the operation and one patient had an internal iliac artery pseudoaneurysm 6 months after the operation. Both patients underwent interventional procedures to seal the aneurysm with an membrane-covered stent, and the former died from aneurysm rupture and bleeding due to persistent infection with multi-drug resistant Pseudomonas Aeruginosa in blood and urine, while the latter had a better interventional outcome with normal renal function. In addition, one pediatric renal transplantation recipient had an abrupt decrease in urine output 2 days after surgery, with ultrasound suggesting poor perfusion, and exploratory surgery of the transplanted renal revealed a kinking in the renal artery, and the renal was removed because renal function could not be restored.

**Table 1:** Vascular complications after renal transplantation.

Complications	*n*	Onset of disease	Therapeutic options	% (% total*)
Renal artery stenosis	36	3.7 ± 2.7 months	Balloon catheter dilation (*n* = 3), Endovascular stenting (*n* = 33)	1.56
External iliac artery dissection	5	<1day (*n* = 4), 3 months (*n* = 1)	Artificial revascularization (*n* = 3), Donor iliac artery bypass (*n* = 1), Interventional placement of membrane-covered stent (*n* = 1)	0.22
Renal artery disruption	4	14.5 ± 2.5 days	Nephrectomy (*n* = 3), Repatch (*n* = 1)	0.17
Renal vein thrombosis	3	11.0 ± 3.6 days	Nephrectomy	0.13
Renal artery thrombosis	2	7 days	Nephrectomy (*n* = 1), PTA, Thrombolysis (*n* = 1)	0.09
Renal artery dissection	1	3 days	PTA	0.04
Renal artery kinking	1	2 days	Nephrectomy	0.04
Pseudoaneurysm of the internal iliac artery	1	6 months	PTA	0.04
Pseudoaneurysm of the renal artery	1	3 months	PTA	0.04
Total	54	—	—	2.34

* Rate of vascular complications to the total number of 2,304 renal transplantations.

## Discussion

The 21st century is known as the era of organ transplantation, and organ transplantation is currently recognized as the most effective treatment for end-stage organ failure, of which renal transplantation is the earliest, most numerous, and most successful organ transplantation. Although the incidence of post-transplant vascular complications is not high, improper treatment can still cause irreversible damage to the function of the transplanted renal and even endanger the life of the patient. Therefore, early detection, early diagnosis, and early treatment of post-transplant vascular complications are very crucial (4). Digital subtraction angiography (DSA) is the gold standard for diagnosing vascular complications. From this study, we can summarize the etiology of vascular complications after renal transplantation into the following causes (5–9), (1) Recipient factors, commonly in advanced age, history of diabetes mellitus, history of atherosclerosis and heart disease, history of hypertension, cytomegalovirus (CMV) infection, a sudden increase in abdominal pressure, history of vascular trauma or surgery on the operative side, hypercoagulatory state, etc. (2) Donor factors, donor age >50 years old, marginal donor, donor renal artery lesion, donor-derived infection (DDI), etc. (3) Transplantation-related factors, DGF, cold ischemia time of the donor's kidney >24 h, immune induction protocol, rejection reaction, etc. (4) Surgery-related factors, donor artery intimal damage caused during donor renal acquisition, poor vascular anastomosis technique, postoperative scar contracture, improper placement of the transplanted renal, compression of the renal artery after the mechanization of the surrounding hematoma, angulation or distortion of the artery, etc. Since January 1, 2015, the Chinese government has completely stopped using organs from executed prisoners, and DD has become the only source of organs other than living organ transplantation (10). However, unfavorable factors such as marginal donor kidney in the era of DD (11), donor underlying diseases leading to vascular lesions, DDI (12), prolonged cold and heat ischemia time, the increased incidence of DGF have increased the potential risk of occurrence of vascular complications (13). To reduce the occurrence of vascular complications after renal transplantation, the focus is on prevention, and we suggest that adequate preoperative assessment of donor-recipient high-risk factors, reasonable selection of vascular segments, gentle intraoperative operations, improved vascular suturing techniques, and targeted postoperative prevention can prevent or reduce the occurrence of such vascular complications.

**Figure 1:**
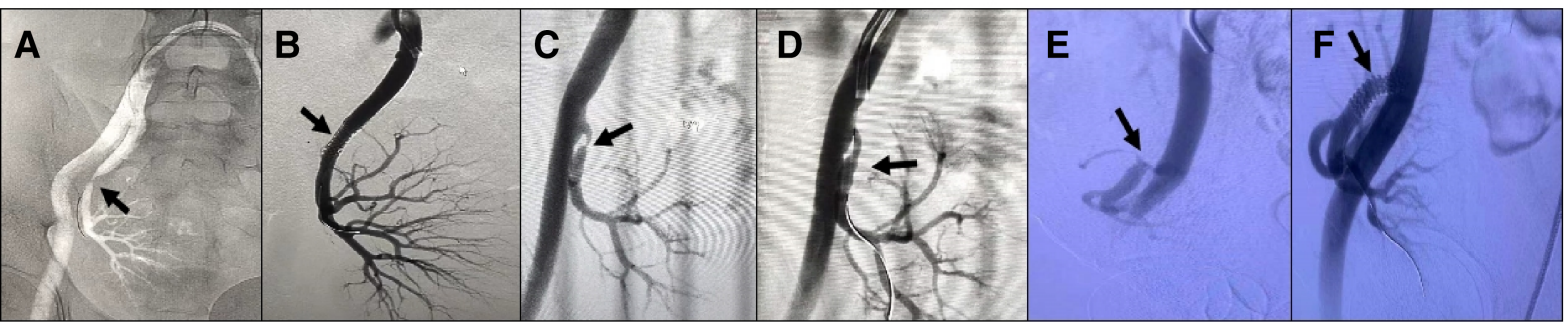
Different treatment modalities for common types of transplanted renal artery stenosis. (**A,B**) Patient 1, male, 44 years old, 5 months after renal transplantation, DSA: Severe stenosis in the middle segment of the transplanted renal artery with 90% stenosis, placement of an arterial stent, normalization of blood supply and renal function of the transplanted renal. (**C,D**) Patient 2, female, 12 years old, 3 months after renal transplantation (child donor kidney 4 years old), serum creatinine (Scr) 270 umol/l, DSA: Anastomosis of external iliac artery, stenosis at 0.5 cm of the distal end of anastomosis with 80% stenosis, and balloon pressure should not be too high, subsequent dilatation may be required. The blood supply of the transplanted renal returned to normal on postoperative review, and the Scr was reduced to 90 umol/l. (**E,F**) Patient 3, male, 3 years and 8 months after renal transplantation, decreased urine output, Scr 350 umol/l, DSA: External iliac artery anastomosis, stenosis at the anastomosis of the transplanted renal artery, 90% stenosis, placement of an arterial stent, normalization of the blood supply of the transplanted renal, Scr decreased to 150 umol/l.

TRAS is the most common vascular complication after renal transplantation, often occurring 3–6 months after surgery, with an incidence of about 1%–23% (14, 15), and the incidence of TRAS in this study was 1.56%. The clinical manifestations of TRAS are refractory hypertension or new hypertension, impairment of graft kidney function with oliguria and edema, and new vascular murmur on auscultation in the graft kidney (16). PTA mainly includes balloon catheter dilatation and endovascular stent-toplasty, the former is suitable for mild TRAS patients, postoperative stenosis rate of 13%–25%, the latter is considered a better treatment of TRAS, also having stent placement failure, renal artery dissection, artery tear risk (17). If intervention is unsuccessful, the stenotic segment can be surgically removed and the artery reanastomosed. Currently, our preferred method for the treatment of TRAS is PTA, which includes 33 cases of successful placement of renal artery stents, 3 cases of balloon dilatation, and no patients who underwent open surgical treatment (Figure 1). 3 patients in our study developed stenosis again, one was a pediatric renal transplantation recipient who underwent balloon dilation again, and two had renal artery stents repositioned. 36 patients with stenosis of the transplanted renal artery had normalized or significantly improved renal function after the intervention, and the overall treatment outcome was satisfactory.

**Figure 2:**
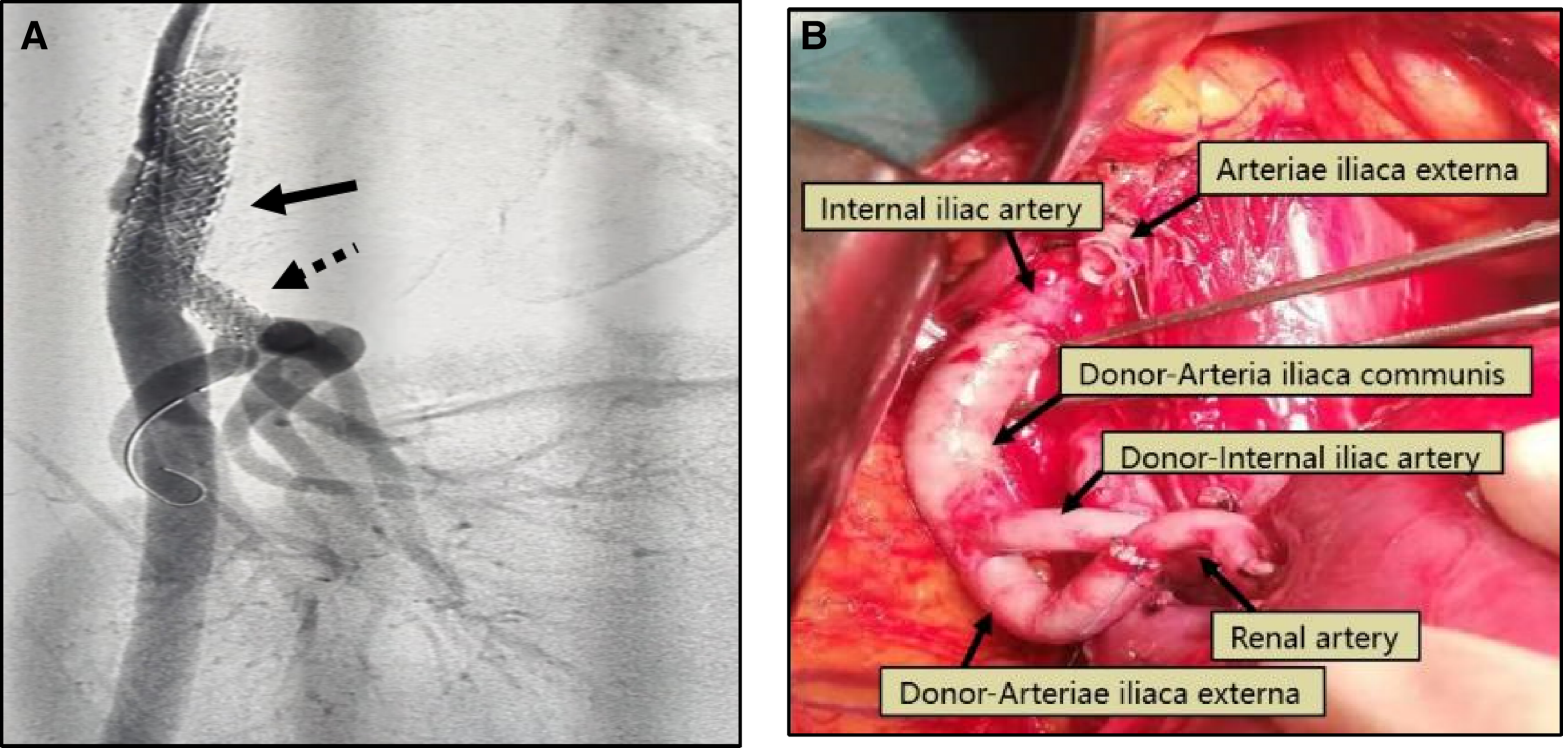
(**A**) Patient 4, male, 42 years old, 3 months after renal transplantation, decreased urine output, Scr 382 umol/l, DSA: external iliac artery dissection (black arrow) and stenosis of the transplanted renal artery (black dotted arrow), membrane-covered stent implanted in the external iliac artery, balloon dilated stent placed in the transplanted renal artery, postoperative Scr decreased to 135 umol/l. (**B**) Patient 5,male, 48 years old, intraoperative external iliac artery dissection occurred, removed the dissection part of the vessel, ligated the proximal end of the external iliac artery, isolated the recipient internal iliac artery, ligated the distal end, used the donor common iliac artery to anastomose with the proximal end of the internal iliac artery, the donor external iliac artery to anastomose with the transplanted renal artery, and the donor internal iliac artery to anastomose with the distal end of the recipient external iliac artery. 5 days after surgery, renal function returned to normal.

External iliac artery dissection is a serious vascular complication that has a low incidence and must be given adequate attention, with a reported incidence of 0.3% in the literature (18), often occurring intraoperatively or within days to months of renal transplantation. Once intraoperative external iliac artery dissection is clearly diagnosed or highly suspected, immediate and prompt management should be made to minimize further progression of the dissection and to protect the blood supply to the transplanted renal and lower extremities. There is no definite treatment for intraoperative external iliac artery dissection in renal transplantation, but currently, the main treatment includes artificial vessel replacement, interventional treatment, and donor artery transplantation (19–21) (Figure 2). Artificial vessel replacement is suitable for sudden and severe intraoperative external iliac artery dissection, which can solve the dissection problem quickly and efficiently, but the cost is relatively high; interventional treatment is mostly used for postoperative external iliac artery dissection, which is less invasive and safer, and the technical difficulty is to distinguish between true and false lumen, and the main disadvantages are contrast agent nephrotoxicity and arterial puncture injury; donor artery graft indications are similar to the artificial vascular replacement, with better histocompatibility, but it is necessary to obtain the organ with a reserved vessel of the same caliber or a hospital with a vascular bank (22). This study was performed in four patients with sudden intraoperative softening of the grafted kidney, decreased urine output, and decreased pulsation of the external iliac artery, and decreased pulsation of the graft artery in 3 cases and disappeared pulsation of the graft artery in one case. 3 cases were treated with artificial vessel replacement and one case was treated with donor iliac artery bypass, and all of the graft eventually returned to normal function.

**Figure 3:**
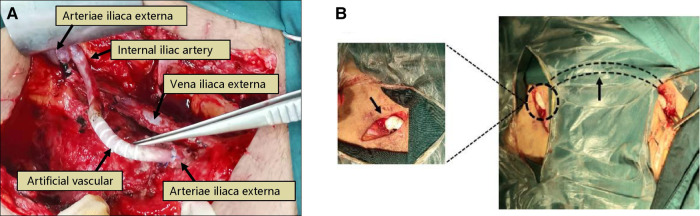
Treatment of transplanted renal artery rupture. Patient 6, male, 39 years old, 13 days after renal transplantation, presented with wound pain and increased drainage, intraoperative rupture of the anastomosis of the transplanted renal artery was seen, and there were signs of infection on the wound surface, considering “infectious arteritis” causing rupture. The renal and part of the external iliac artery were removed, the proximal end of the external iliac artery was ligated, and the proximal end of the internal iliac artery was anastomosed with an artificial vessel (Poly tetra fluoroethylene, PTFE), (**A**) 7 days after surgery, the bleeding reoccurred, and intraoperative bleeding was seen at the anastomosis between the artificial vessel and the distal end of the external iliac artery. The stump of the distal end of the external iliac artery and the proximal end of the internal iliac artery was ligated, and a femoral artery-artificial vessel-contralateral femoral artery bypass was performed, (**B**).

The rupture of the renal artery or vein due to surgical factors usually occurs within 2 weeks after surgery, and the rupture of the vessel due to infection is usually observed 2–4 weeks or more after surgery, which often leads to graft loss or even death of the recipient (23, 24). The onset of renal artery rupture is rapid, vicious, and life-threatening, so once diagnosed, it should be actively managed and prepared for surgery. Surgical exploration should be handled according to the etiology of the ruptured vessel (25, 26): (1) Anastomotic leak caused by poor suturing of the vessel, the vascular repair is feasible; (2) Rupture of the vessel caused by infectious arteritis, the transplanted renal should be removed; (3) For recipients with external iliac artery anastomosis of the transplanted renal artery, the proximal and distal ends of the infected site of the external iliac artery should be ligated after removal of the transplanted renal, the infected site should be left open, and the distal end of the vessel should undergo femoral artery-artificial vessel-contralateral femoral artery bypass surgery or ipsilateral axillary artery-artificial vessel-femoral artery bypass surgery to restore blood supply to the ipsilateral lower limb (Figure 3). 4 cases of renal artery rupture occurred in this study, all in the perioperative period, 3 cases were diagnosed as ruptured due to infectious arteritis and required graft nephrectomy due to irreparable and infected condition, one case was repaired due to loose suture and the function of the transplanted renal was restored to normal.

**Figure 4:**
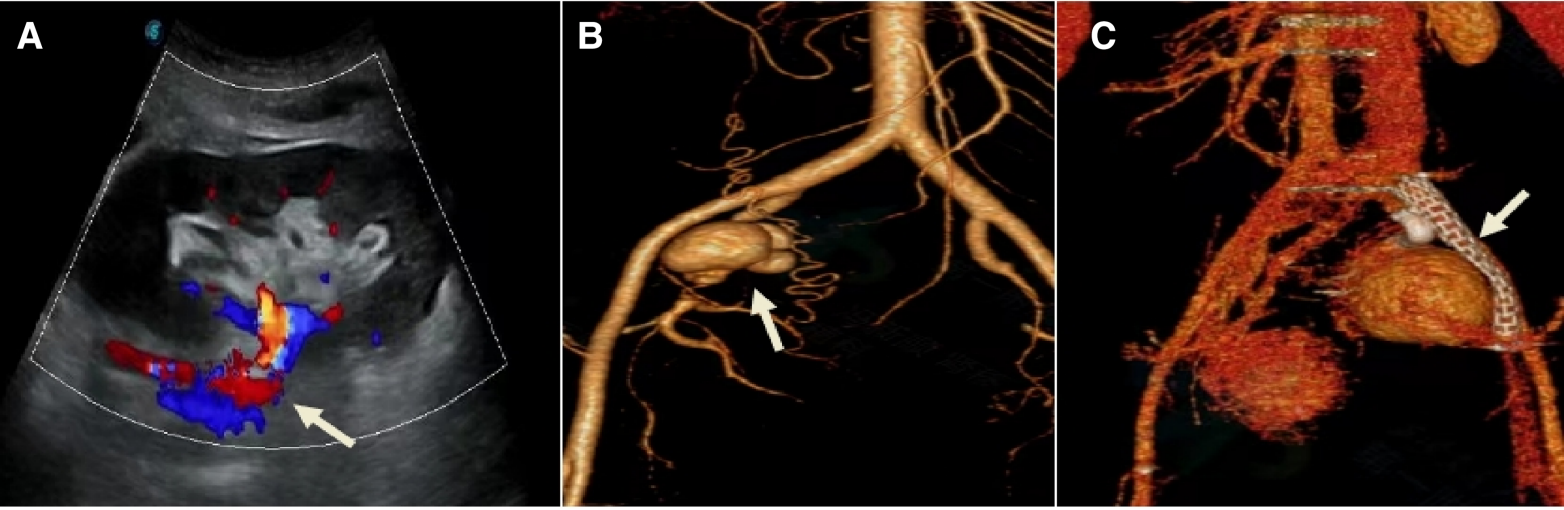
Treatment modality of the transplanted renal aneurysm. Patient 7, female, 34 years old, 2.5 months after renal transplantation, oliguria for 1 day, Scr 354 umol/l. (**A**) Ultrasound of the transplanted renal before admission: Poor perfusion of the renal, with an anechoic area behind the right external iliac artery, about 32 mm × 36 mm in size, connected to the renal artery, with a channel opening of about 6 mm in width. (**B**) DSA: Multiple aneurysmal protrusions were seen in the transplanted renal artery. (**C**) One Viabahn 187 mm–50 mm covered stent was placed so that its distal end was anchored to the main stem of the graft renal artery and the proximal end was anchored to the proximal end of the right internal skeletal artery after release.

Renal aneurysms are rare, and the clinical manifestations of smaller aneurysms are usually asymptomatic. Excessive traction of the renal pedicle during kidney acquisition or intubation perfusion during repair leads to injury to the renal artery, poor vascular anastomosis technique leads to partial opening of the arterial anastomosis to form a pseudoaneurysm, and surgical site infection, especially fungal infection, may lead to the occurrence of renal aneurysms. When the aneurysm increases or ruptures, some patients may experience pain, swelling, perirenal hematoma, hypertension, hematuria, and abnormal graft function, leading to hemorrhagic shock and life-threatening conditions (27). Conservative treatment can be chosen for aneurysms that have not yet caused changes in renal hemodynamics and renal function. If the aneurysm has already ruptured or is at high risk of rupture, interventional procedures for renal artery embolization, implantation of covered stents, or open surgical repair may be performed (28). If the renal aneurysm is caused by infection and the risk of rupture is high, a transplanted nephrectomy is feasible. One patient in this study had a renal artery pseudoaneurysm at 3 months after the operation and one patient had an internal iliac artery pseudoaneurysm at 6 months after the operation. Both patients underwent interventional procedures to place covered stents to seal the aneurysm, and the former died because of persistent infection with multi-drug resistant Pseudomonas Aeruginosa in the blood and urine after 1 month of interventional treatment, resulting in rupture and bleeding of the aneurysm, Figure 4. The latter had a better interventional outcome with normal renal function.

Renal vascular thrombosis is an extremely severe complication and if left untreated, it usually leads to graft loss and is one of the major causes of early postoperative transplanted renal loss (29). The incidence of transplanted renal thrombosis ranges from 0.3% to 6.1% and occurs mostly in the first 2 weeks after transplantation (30). Renal artery thrombosis is usually caused by rejection, severe renal artery stenosis, pediatric renal transplantation, and hypercoagulable state (31), while renal vein thrombosis is usually due to external compression caused by proximal deep vein thrombosis in the lower extremity or perigraft hematoma or fluid accumulation. The clinical signs of thrombosis include sudden oliguria or anuria, elevated SCr, occasional graft pain, and diminished or absent renal artery blood flow as seen on ultrasound. We observed in our series 3 patients with renal vein thrombosis and one patient with renal artery thrombosis who underwent surgical exploration and were unable to restore graft function, so the kidney was removed. One patient who developed renal artery thrombosis was treated with thrombolysis and intraluminal stent placement and developed DGF after the operation, and renal function returned to normal after 2 weeks.

Arterial or venous kinking can result from inappropriate activity, vasoconstriction, or large pelvic spaces. If not thrombosed, it is predicted that the kidney graft could be salvaged by surgical exploration / reposition of the kidney graft or other technique to correct the kinking, and PTA treatment is not ideal, which may increase the risk of vasospasm, rupture, or dissection (31). A pediatric renal transplantation recipient in our study series had an abrupt decrease in urine output 2 days after the operation, with ultrasound suggesting poor perfusion, and a renal artery kinking was found during exploratory surgery of the transplanted renal, which may have occurred for reasons related to excessive activity, large incision space leading to the kinking of the transplanted renal artery, renal artery thrombosis and graft nephrectomy. We suggest that the blood vessels retained should not be too long, the incision space be moderate, and the postoperative activity should not be strenuous, the adipose tissue on the surface of the kidney was suture fixed with the incision tissue can reduce the occurrence of vascular kinks.

In this study, 40 cases were treated by PTA, 14 cases were treated by open surgery, and finally 9 patients had graft nephrectomy, with an overall treatment rate of 83.3% (45/54). Most of the late-stage vascular complications can achieve more satisfactory clinical results through PTA. For example, transplanted renal artery stenosis, which mostly occurs 3–6 months after surgery, while all the 36 patients in this study were able to got stenosis relieved through interventional procedures, and the function of the transplanted renal has returned to normal or improved significantly. However, complications such as renal artery rupture, thrombosis, and renal artery kinking are common in the perioperative period and require open surgical treatment due to the presence of thrombosis, infection, and hemorrhage, and the overall treatment outcome is poor, with a transplanted renal loss rate of 64.3% (9/14), requiring focused prevention and attention.

A comprehensive understanding of the etiology, clinical features, and clinical manifestations of post-renal transplantation vascular complications should be given for targeted prevention, and timely detection. A definitive diagnosis, and decisive treatment are the keys to successful management and rescue. Of course, the incidence of vascular complications after renal transplantation can be minimized by each step of the surgeon's delicate operation during the surgery. Considering the small sample size of this study and the fact that some of the complications are reported on a case-by-case basis and the follow-up period is relatively short, further statistical analysis of risk factors, the survival rate of the transplanted renal and other complications could not be gathered, so long-term follow-up is still needed to assess the prognosis.

## Data Availability

The original contributions presented in the study are included in the article, further inquiries can be directed to the corresponding author.
